# Lessons of Macrophage-Associated Heart Regeneration in Fish, Amphibians, and Neonatal Mice, Applied to Adult Mice: A Perspective on α-Gal Nanoparticles

**DOI:** 10.3390/ijms27041950

**Published:** 2026-02-18

**Authors:** Uri Galili, Gary L. Schaer

**Affiliations:** Department of Medicine, Rush University Medical Center, Chicago, IL 60612, USA; gary_schaer@rush.edu

**Keywords:** injured myocardium regeneration, anti-Gal antibody, α-gal epitopes, α-gal nanoparticles, pro-regenerative macrophages

## Abstract

An ancient evolutionary regenerative mechanism of injured myocardium in vertebrates has been conserved in zebrafish, urodeles (salamander, newt, and axolotl) and neonatal mice. This innate regenerative mechanism is characterized by extensive migration of pro-regenerative macrophages into the injured myocardium and non-immune activation of parts of the complement system. Loss of regenerative activity in neonatal mice within a few days after birth implies that it is suppressed and replaced by fibrotic repair and scar formation. Fibrosis prevents ventricular wall rupture following myocardial infarction (MI), but it compromises contractility and can lead to heart failure and premature death. Reactivation of the suppressed regenerative mechanism in post-MI adult mice may be feasible by localized immune activation of the complement system, resulting in extensive recruitment of pro-regenerative macrophages into the injured myocardium, recapitulating neonatal mechanisms. Localized complement activation can be achieved by a new method of harnessing the natural anti-Gal antibody, which constitutes ~1% of human immunoglobulins and binds the carbohydrate antigen “α-gal epitope”. α-Gal nanoparticles (small liposomes presenting multiple α-gal epitopes) bind anti-Gal when administered into reperfused myocardium post-MI in anti-Gal-producing mice, thereby inducing localized complement activation. In this novel approach, macrophages recruited into the ischemic myocardium by complement cleavage chemotactic peptides, and binding anti-Gal-coated α-gal nanoparticles, polarize to become pro-regenerative macrophages that produce pro-regenerative cytokines and recruit stem cells. This process results in near-complete regeneration of the injured myocardium within 14 days. Future evaluation of this novel approach in larger animal models will help in determining whether trans-endocardial delivery by catheter of α-gal nanoparticles into ischemic myocardium warrants clinical application in acute MI.

## 1. Introduction

Heart injuries in adult fish, amphibian urodeles (salamander, newt, and axolotl) and neonatal mice are repaired by regenerative mechanisms that restore native cardiac structure and function. In contrast, myocardial infarction (MI) in adult mammals and in humans results in tissue repair dominated by fibrosis and scar formation. If MI in humans is not promptly treated with reperfusion of the occluded coronary artery, cardiomyocytes die and are permanently replaced by fibrotic tissue forming a scar which prevents rupture of the ischemic ventricular wall. The loss of many cardiomyocytes impairs the contractile function of the heart, which can lead to heart failure and premature death.

The existence of innate myocardial regeneration in fish, urodeles and neonatal mice suggests that better understanding of the common processes of regeneration in these vertebrates may provide clues to activation of postulated suppressed regenerative capability in adult mammals, ultimately leading to improved treatment for patients with MI. This hypothesis has motivated studies to identify regeneration-associated genes activated during heart repair in zebrafish, axolotl and neonatal mice, and determine whether similar gene networks can be reactivated therapeutically in adult mammalian myocardium. This review presents an alternative regenerative strategy by developing a method for inducing macrophages to polarize into “pro-regenerative” macrophages which mediate regeneration of injured myocardium. As described below, pro-regenerative macrophages differ from “pro-reparative” macrophages which mediate the default repair mechanism of fibrosis and scar formation in adult mammals post-MI. The rationale for studying macrophages for regeneration induction in adult mammals is that extensive infiltration of macrophages into injured myocardium is one of the first events observed in the process of myocardial regeneration in all three vertebrate models displaying innate heart regeneration. These innate regeneration processes are described in the first part of this review and are contrasted with the default fibrosis and scar formation repair in injured myocardium in adult mice. The second part describes the method for generation of pro-regenerative macrophages by α-gal nanoparticles. These macrophages mimic the activity of pro-regenerative macrophages in fish, urodeles and neonatal mice, by inducing scar-free regeneration of injured tissues in adult mice. The third part reviews experimental evidence of near-complete regeneration of adult mouse post-MI myocardium following α-gal nanoparticle-mediated immune modulation.

## 2. Macrophages Mediating Heart Regeneration in Fish

Zebrafish possess a robust capacity for cardiac regeneration following injury. Surgical resection of the ventricular apex triggers cardiomyocyte proliferation, resulting in restoration of myocardial structure and function without fibrosis or scar formation, a response that contrasts sharply with adult mammalian heart repair [1,2,3]. Transcriptomic analyses reveal activation of numerous regeneration-associated genes, including more than 135 transcription factors that collectively orchestrate this complex regenerative response [4,5,6]. The cellular origin of regenerated cardiomyocytes remains under investigation. Regeneration of the injured myocardium in zebrafish may involve proliferation of residential cardiomyocytes activated by appearance of various transcription factors, recruitment and differentiation of stem cells/progenitor cells, or a combination of both. Regardless of cellular origin, the regeneration process of the injured myocardium is associated with the activity of distinct populations of pro-regenerative macrophages [7,8,9] which seem to interact with various types of cells participating in the regeneration process [10]. Accordingly, elimination of macrophages by clodronate at the time of heart injury in zebrafish was found to prevent myocardial regeneration [11,12]. Phenotyping of macrophages suggests that they are present at different activation states which are associated with their ability to induce regeneration or fibrosis and scar formation [8]. The factors determining polarization of macrophages to pro-regenerative or pro-reparative cells have yet to be determined.

In addition to macrophages participating in zebrafish heart regeneration, a second component of the immune system was found to be activated in this regenerative process. This component is part of the complement system. Resection of 10% to 20% of the ventricular mass of the zebrafish heart was found to be followed by elevated expression of the genes of complement 5a receptor 1 (*C5AR1* gene) and complement 3a receptor 1 (*C3AR1* gene) in the ventricular wall [13]. Accordingly, inhibition of C5aR1 activation by the C5aR1 antagonist PMX205 was found to significantly attenuate the cardiomyocyte proliferative response. These findings implied that activation of the complement receptors C5aR1 and C3aR1 is necessary for the complex heart regeneration process in zebrafish. As discussed below, similar complement-dependent mechanisms operate during cardiac regeneration in axolotl and neonatal mice, suggesting evolutionary conservation [13].

## 3. Macrophages in Heart Regeneration in Urodeles

Urodeles represent the most extensively studied vertebrate model of tissue regeneration. The seminal observation by Lazzaro Spallanzani in 1768 that salamanders can regenerate amputated limbs established the field of regenerative biology. Subsequent studies in axolotl demonstrated that regeneration depends critically on early macrophage recruitment to the injury site. These macrophages mediate formation of the blastema, a proliferative cell mass essential for limb regrowth [14]. Macrophage depletion prevents blastema formation and redirects healing toward fibrotic repair without regeneration [14]. Cardiac regeneration in urodeles follows analogous principles. Adult newt hearts regenerate following injury through cardiomyocyte proliferation without fibrosis or scarring [15]. As in limb regeneration, salamander heart regeneration requires early macrophage infiltration [16]. Among their functions, regenerative macrophages secrete metalloproteinases that remodel extracellular matrix, thereby facilitating cell migration and tissue restructuring. Temporal studies indicate that macrophages are required during early regenerative phases in urodeles, but are dispensable once cardiomyocyte proliferation is established, revealing stage-specific roles in regeneration [16]. Complement activation also contributes to urodele cardiac regeneration. The upregulation of the complement *C5AR1* and *C3AR1* genes observed in regenerating zebrafish heart, was also found in early stages of regeneration in the axolotl heart [13]. In addition, treatment of axolotl with the C5aR1 antagonist PMX205 resulted in prevention of effective regeneration, like that observed in zebrafish. These findings suggest similarity in the contribution of macrophage and complement system components to heart regeneration in both vertebrates.

## 4. Regeneration of Injured Heart in Neonatal Mice

The most compelling evidence supporting conservation of cardiac regenerative capacity in mammals derives from studies in neonatal mice. During the first days of life, neonatal mice regenerate cardiac injuries, including ventricular apex resection, in a manner comparable to zebrafish and urodeles [17,18]. Similar regenerative competence has been reported in neonatal pigs [19,20]. This capacity is transient; in mice older than 7 days, identical injuries heal through fibrosis and scar formation, recapitulating adult mammalian responses [17,18]. The regeneration of injured heart in neonatal mice is initiated by rapid infiltration of macrophages into the injury site, followed by extensive cardiomyocyte proliferation [21,22]. These processes parallel regenerative mechanisms in zebrafish and urodele adults. Complement activation is also conserved, with upregulation of C5aR1 and C3aR1 in injured neonatal mouse myocardium [13]. It is of interest to note that regenerative competence in early mammalian life is not restricted to the heart. Mouse embryos heal cutaneous wounds without scarring [23], whereas adult mice exhibit fibrotic repair. Collectively, these observations suggest that zebrafish and urodele regeneration represent an evolutionary ancient program retained in mammals but suppressed shortly after birth. This concept has given rise to the hypothesis that the “cardiac regenerative clock” may be reversible [24]. As described below, the two immune-associated phenomena, macrophage recruitment and complement system activation, observed in the innate heart regeneration of fish, urodeles and neonatal mice, can be induced by α-gal nanoparticles which bind the anti-Gal antibody in adult mice [25] and lead to post-MI regeneration of injured myocardium.

## 5. Macrophages in Repair of Injured Heart in Adult Mice

**Figure 1:**
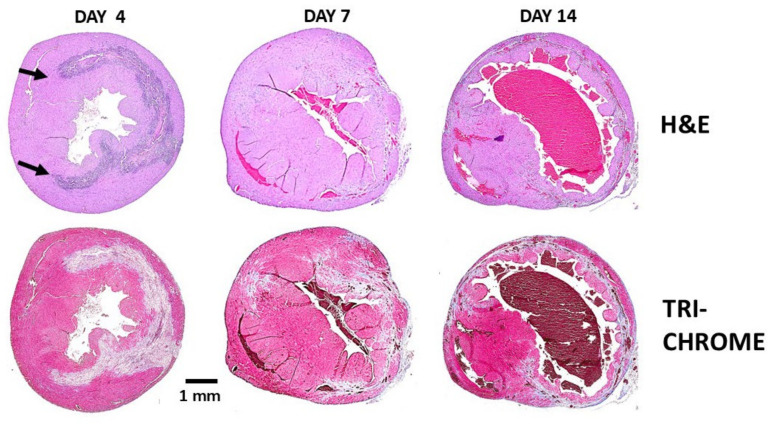
Representative post-MI repair of the left ventricular wall in adult mouse heart treated with saline, was monitored for 2 weeks. The sections are stained with H&E (macrophages as purple dots) or Mason Trichrome staining (cardiomyocytes—red, fibrosis—grey and blue, and debrided areas—light pink or white). Day 4: Macrophages migrate into the ischemic myocardium and debride the injured tissue. The edges of the infiltration are marked by arrows. Day 7: Most of the infiltrating macrophages disappear from the debrided areas. Day 14: The injured myocardium undergoes fibrosis; the myocardial wall is thinning and converted into scar tissue. Adapted from Ref. [34] with permission.

The post-MI repair of injured myocardium in adult mammals, including that of adult mice and humans differs from that observed in zebrafish, urodeles and neonatal mice. Repair of injured myocardium in adult mammals is mediated by the mechanism of fibrosis and scar formation which prevents rupture of the injured ventricular wall. But the irreversible fibrosis of the injured wall also results in reduced contractility which can lead to heart failure and premature death [26]. Experimental studies of post-MI repair in mouse heart reveal parallels with cutaneous wound healing [27,28,29,30]. In both tissues, early infiltration of pro-inflammatory macrophages facilitates clearance of necrotic debris. This phase is followed by accumulation of pro-reparative macrophages that secrete cytokines promoting angiogenesis, fibroblast activation, extracellular matrix deposition, and scar formation. These sequential events are illustrated in Figure 1 using H&E staining at defined time points following mid-left anterior descending (LAD) coronary artery occlusion for 30 min, followed by reperfusion. The same sections also were stained by Mason Trichrome (Figure 1) for identifying fibrotic tissue, stained gray or blue, and the myocardium, stained red. Extensive infiltration of macrophages is observed after 4 days in H&E staining, and elimination of injured myocardial cells is indicated by “empty” debrided areas, which are likely to be the result of proteolytic activity mediated by proteinases secreted by the macrophages. After 7 days most of the macrophages disappear and within 14 days the injured ventricular wall undergoes thinning and fibrosis.

The observations on cardiomyocyte proliferation in the injured neonatal mouse heart led to the assumption that methods for activation of adult mouse cardiomyocytes may help in the induction of regeneration of injured adult mouse heart. Motivated by this rationale, therapeutic methods for inducing cardiomyocyte proliferation to achieve regeneration of injured myocardium in adult mammals were studied. Many of these methods are summarized in references [31,32,33] and range from oxygen level modulation and energy metabolism to regulation of cell-cycle genes, hormone administration, administration of various growth factors and their genes, genes associated with signaling pathways, transcription factor genes, and extracellular matrix hydrogel.

The solution for inducing regeneration in adult mouse heart presented in this review differs from the other approaches described in references [31,32,33] in that it aims to modulate the macrophages migrating into the injury sites. In such a modulation, the default repair mechanism of fibrosis and scar formation is replaced by regeneration of the normal structure and function of the injured myocardium [34]. Based on the regenerative outcomes of their activity, these macrophages are referred to as “pro-regenerative macrophages” since they display similar activities to those mediating regeneration in fish and amphibian urodeles [35,36,37]. These macrophages contrast “pro-reparative macrophages” mediating the default repair in adult mammals by fibrosis and scar formation, presented in Figure 1 [26,27,28,29,30].

The manipulation of macrophages to polarize into pro-regenerative macrophages was achieved by the novel technology of harnessing the natural anti-Gal antibody with α-gal nanoparticles which are α-gal liposomes having an average size of ~300 nm [38,39,40].

## 6. Anti-Gal/α-Gal Nanoparticle Interaction Recruits Pro-Regenerative Macrophages and Stem Cells

Two of the common characteristic processes in regeneration of injured heart in zebrafish, urodeles and neonatal mice are the extensive infiltration of macrophages and activation of complement receptor C5aR1 and C3aR1 in the injured area [13]. Similarly, the C3 and C5 components of the complement system were found to be essential in newt limb and lens regeneration [41] and even in the regeneration process of injured mouse liver [42,43]. In view of these characteristics, it was of interest to determine whether localized activation of the complement system, achievable in humans by antigen/antibody interaction, can contribute to modulation of recruited macrophages into pro-regenerative macrophages.

An antigen/antibody interaction that is applicable to all humans must rely on an antibody that is naturally abundant in humans—one that is produced throughout life at high titers and has well-defined antigenic specificity. The antibody chosen for this purpose is the natural anti-Gal antibody, constituting ~1% of human immunoglobulins [25,44,45,46,47,48]. Anti-Gal binds specifically to the carbohydrate antigen (ligand), the α-gal epitope with the structure Galα1-3Galβ1-4GlcNAc-R [25,49,50,51]. Anti-Gal is produced throughout life in humans [44] as IgG, IgM and IgA isotypes [52,53] because of constant antigenic stimulation by gastrointestinal bacteria presenting antigens with structures like that of the α-gal epitope [54,55,56,57]. The α-gal epitope is synthesized on glycolipids and glycoproteins of non-primate mammals, lemurs and New-World monkeys by the glycosylation enzyme α1,3galactosyltransferase (α1,3GT) [58,59,60,61], which is encoded by the *GGTA1* gene [62,63]. *GGTA1* is inactive in Old-World monkeys, apes and humans [64,65,66], all lacking α-gal epitopes and producing the natural anti-Gal antibody [61,67,68].

Binding of the natural anti-Gal antibody to α-gal epitopes was found to generate immune complexes that induce potent activation of the complement system, resulting in cytolysis of mammalian cells presenting α-gal epitopes [69,70,71,72,73]. This extensive anti-Gal/α-gal epitope-mediated complement activation was demonstrated in vivo in xenotransplantation studies in which porcine heart or kidney were transplanted in Old-World monkeys. These xenografts were rejected within 30 min to several hours due to activation of the complement system by the recipient’s anti-Gal binding to multiple α-gal epitopes on the endothelial cells lining the blood vessels within a pig xenograft [70,74,75,76].

**Figure 2:**
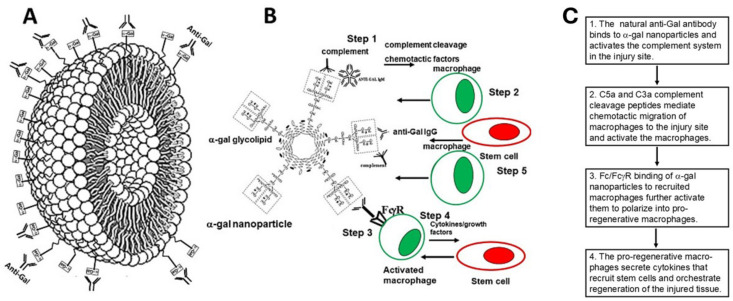
Illustration of a biodegradable α-gal nanoparticle (**A**) and of the hypothesized processes (steps) occurring following anti-Gal binding to α-gal nanoparticles (**B**,**C**). (**A**) The α-gal nanoparticle is a small liposome with a phospholipid wall and multiple α-gal epitopes (rectangles) capping glycolipids. The α-gal epitopes on the nanoparticles readily bind anti-Gal when introduced into humans or into animals producing the anti-Gal antibody. The nanoparticle wall also contains cholesterol molecules which stabilize the nanoparticles. (**B**) Steps following administration of α-gal nanoparticles into injuries. Step 1: Anti-Gal binding to α-gal epitopes on the nanoparticles locally activates the complement system, which generates the complement chemotactic cleavage peptides C5a and C3a. Step 2: The chemotactic peptides C5a and C3a direct extensive migration of macrophages to the site of the α-gal nanoparticles. Step 3: The recruited macrophages bind α-gal nanoparticles via the interaction between the Fc portion (“tail”) of anti-Gal IgG immunocomplexed to the nanoparticles and Fcγ receptors (FcγR) on the recruited macrophages. Step 4: The macrophages binding α-gal nanoparticles are activated and polarize into pro-regenerative macrophages, which secrete a variety of regenerative cytokines. Step 5: Among the secretions of the activated macrophages there are cytokines that recruit stem cells to the site of the α-gal nanoparticles. (**C**). Flow chart of macrophage activation by α-gal nanoparticles. Adapted from Ref. [83] with permission.

The observations on complement activation by anti-Gal/α-gal epitope interactions led to development of biodegradable liposomes (size ≥500 nm) or of smaller liposomes (size ~300 nm), referred to as ”α-gal nanoparticles” which present multiple α-gal epitopes. It was hypothesized that administration of these liposomes/nanoparticles into an anti-Gal-producing mammal will result in anti-Gal binding to them followed by localized activation of the complement system [38,39]. Rabbit red cell membranes were chosen as a biological source for α-gal liposomes production because these red cells present several fold more α-gal epitopes than all other mammalian red cells tested [67]. Production of α-gal liposomes/nanoparticles includes overnight extraction of a mixture of α-gal glycolipids, phospholipids and cholesterol from the rabbit red cell membranes in a chloroform:methanol solution at a 1:2 ratio [38,39]. After filtration for removal of solids, the extract is dried in a rotary evaporator, saline is added to the dried extract, and the suspension is sonicated in a sonication bath for forming α-gal liposomes. Additional sonication of the liposomes with a sonication probe results in conversion of the liposomes into smaller α-gal nanoparticles [39] (Figure 2A). These nanoparticles are biodegradable and can also be produced with synthetic glycolipids capped with α-gal epitopes.

It was hypothesized that administration of α-gal nanoparticles (or α-gal liposomes) to wounds or into injured internal tissues will result in binding of anti-Gal, released from ruptured capillaries to these nanoparticles (Figure 2B) [38,39,40]. Anti-Gal/α-gal nanoparticle interaction will further induce localized activation of the complement system (Step 1) and the generated byproducts, C5a and C3a complement cleavage chemotactic peptides, will induce extensive recruitment of macrophages to the injury site (Step 2) [77,78]. The interaction of increasing concentrations of C5a with C5a receptors (C5aR, a G protein-coupled receptor) on macrophages was found to induce ERK1/2 phosphorylation [79] calcium mobilization and Rho activation [80] leading to chemotaxis toward the site of anti-Gal binding to α-gal nanoparticles and activation of these macrophages. Recruited macrophages will further bind the anti-Gal opsonized liposomes/nanoparticles, by Fc/Fcγ receptor (FcγR) interactions, which activates intracellular signaling that further activates the macrophages and induces phagocytosis of the α-gal nanoparticles through phosphorylation of several signaling pathways (Step 3) [81,82]. Binding of C3b on the anti-Gal-coated α-gal nanoparticles to complement receptor 1 (CR1) further contributes to the phagocytosis and activation of the recruited macrophages. As described above, activation of the complement system and infiltration of macrophages into the injury site are two steps also observed in the innate regeneration in zebrafish, urodeles and neonatal mice. Therefore, it was hypothesized that administration of α-gal liposomes/nanoparticles to injuries in anti-Gal-producing adult animals or humans may induce polarization of the recruited macrophages into pro-regenerative macrophages that can orchestrate regeneration. Such regeneration may be mediated by various cytokines secreted by the pro-regenerative macrophages including VEGF and cytokines recruiting stem cells (Step 4) [39,40].

**Figure 3:**
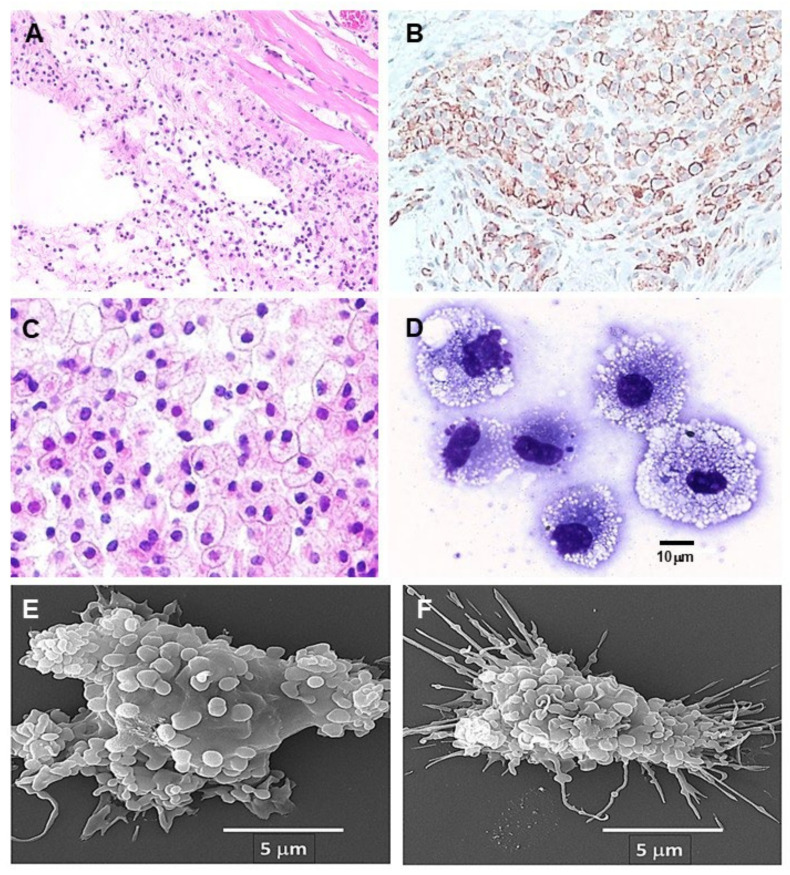
Anti-Gal-mediated recruitment of macrophages and binding of α-gal nanoparticles to macrophages. (**A**) Intradermal injection of 10 mg α-gal nanoparticles in GT-KO mice producing anti-Gal; results within 24 h in extensive recruitment of macrophages to the injection site. The empty areas contained the injected α-gal nanoparticles, which were dissolved by alcohol during the processing of the section (H&E × 100). (**B**) Macrophages recruited within 4 days post-injection (as in (**A**)) and stained with the peroxidase-coupled macrophage-specific anti-F4/80 antibody (×200). (**C**) Peak of macrophage recruitment observed 7 days post-injection. The macrophages are large with ample cytoplasm (×400). (**D**) Macrophages obtained from polyvinyl alcohol (PVA ) sponge discs containing 10 mg α-gal nanoparticles, implanted subcutaneously for 7 days in anti-Gal-producing mice. The many vacuoles within the large macrophages are of the multiple anti-Gal-opsonized α-gal nanoparticles internalized by the macrophage. The nanoparticles were dissolved by alcohol fixation (×1000). (**E**,**F**) Scanning electron microscopy (SEM) of two macrophages incubated for 2 h with anti-Gal opsonized α-gal nanoparticles, and then washed, fixed and processed for SEM. The many small spheres “covering” the macrophages are α-gal nanoparticles that bind to the macrophages via the Fc/Fcγ receptor interaction. Adapted from Ref. [88] with permission.

The five steps illustrated in Figure 2B and detailed in Figure 3C could be demonstrated in knockout mice for the *GGTA1* gene, i.e., mice lacking α-gal epitopes (referred to as GT-KO mice) [84]. These mice can produce anti-Gal following immunization with cells or cell membranes presenting multiple α-gal epitopes, such as rabbit or porcine cells, porcine kidney membrane homogenate, or bacteria [85,86,87,88].

### 6.1. Recruitment of Macrophages by Anti-Gal/α-gal Nanoparticle Interaction (Steps 1–3)- 

The effective chemotactic activity of the complement system following its activation by the anti-Gal/α-gal nanoparticle interaction (Steps 1 and 2 in Figure 2B) could be demonstrated by intradermal injection of α-gal nanoparticles into GT-KO mice producing anti-Gal. Within 24 h, many monocytes extravasated and differentiated into macrophages that surrounded the injection area (Figure 3A). The number of macrophages increased after 4 days, and they were identified by immunostaining with the macrophage-specific anti-F4/80 antibody (Figure 3B). The macrophage recruitment peaked by day 7 (Figure 3C). At that time, the macrophages displayed a large size with ample cytoplasm filled with many vacuoles containing the anti-Gal opsonized α-gal nanoparticles, due to extensive phagocytosis of these nanoparticles (Figure 3C,D). The nanoparticles could not be detected since they were dissolved by alcohol in the staining process. The observed phagocytosis occurred following the interaction between the Fc portion of anti-Gal bound to the α-gal nanoparticles and Fcγ receptors on the macrophages. This interaction (Step 3 in Figure 2B) is demonstrated in Figure 3E,F in which the nanoparticles are seen “covering” the surface of two macrophages following co-incubation of the macrophages, for 2 h at room temperature, with nanoparticles coated (opsonized) by anti-Gal. The recruited macrophages did not form any chronic granuloma, and they completely disappeared after 3 weeks, without affecting the normal structure of the skin [88].

### 6.2. Characterization and Activities of the Recruited Macrophages

**Figure 4:**
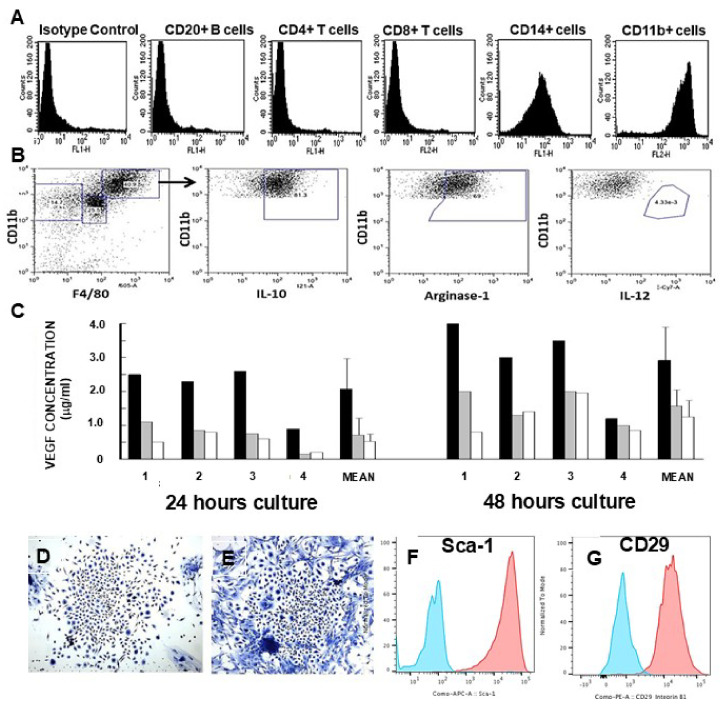
Characteristics of macrophages recruited by α-gal nanoparticles and of recruited stem cells. Cells recruited into PVA sponge discs containing 10 mg α-gal nanoparticles and implanted subcutaneously for 7 days into anti-Gal-producing GT-KO mice. Cells were retrieved from the explanted sponge discs and subjected to various analyses. (**A**) Flow cytometry analysis for various markers. Most of the cells were found to be macrophages as they expressed the macrophage-specific CD11b and CD14 markers, whereas no significant number of cells displayed the CD20 marker of B cells or CD4 or CD8 markers of T cells. (**B**) Analysis of the large macrophages (CD11b^pos^/F4/80^pos^) for M1 or M2 markers of polarized macrophages indicated that most macrophages displayed IL10 and Arginase-1 markers of M2 macrophages, whereas most cells lacked the M1 marker of IL12 production. (**C**) In vitro co-incubation of macrophages for 24 or 48 h with anti-Gal-opsonized α-gal nanoparticles activates the macrophages to secrete VEGF. Macrophages cultured with anti-Gal-opsonized α-gal nanoparticles (closed columns), macrophages cultured with α-gal nanoparticles not opsonized by anti-Gal (gray columns), or macrophages with no α-gal nanoparticles (open columns). Data from 4 GT-KO mice and means +SD. (**D**,**E**) Macrophages as in (**A**,**B**), cultured for 5 days, yielded cell colonies like those produced in cultures of bone marrow stem cells. Each colony grew from a suspension of 50,000 to 100,000 macrophages migrating into the PVA sponge discs. (**F**,**G**) The cells harvested from the colonies as in (**D**,**E**) stained positive in flow cytometry for the stem cell markers Sca-1 and CD29. Isotype control—blue; marker staining—orange. Adapted from Refs. [40,88] with permission.

Characterization of the cells recruited by anti-Gal/α-gal nanoparticle interaction was achieved by implanting the mice subcutaneously with biologically inert polyvinyl alcohol (PVA) sponge discs, containing 10 mg α-gal nanoparticles, for a period of 7 days. Within that period, ~0.5 × 10^6^ macrophages were recruited into these sponge discs. The macrophages retrieved from the explanted PVA sponge discs displayed morphology like that in Figure 3D. Flow cytometry analysis of the macrophages indicated that they displayed the macrophage markers CD11b and CD14, whereas no T cells or B cells were detected among the recruited cells (Figure 4A). Most recruited macrophages were polarized as M2 macrophages, which are positively stained for IL10 and Arginase-1 and negatively stained for IL12 (a characteristic of M1 macrophages) (Figure 4B). In vitro interaction of macrophages with anti-Gal-coated α-gal nanoparticles was further found to induce the macrophages to secrete several cytokines including VEGF (Step 4 in Figure 2B) inducing neo-vascularization (Figure 4C) [39]. In addition, these macrophages secrete cytokines, which recruit stem cells. This was demonstrated by culturing the infiltrating cells for 5 days, resulting in the appearance of cell colonies like cultured stem cell colonies (one colony per 50,000 to 100,000 cultured macrophages) (Figure 4D,E). The cells within these colonies displayed the stem cell markers Sca-1 and CD29 (Step 5 in Figure 2B) (Figure 4F,G) [40]. qRT-PCR studies with macrophages recruited in the skin by intradermal injection of α-gal nanoparticles (as in Figure 2A,B), further demonstrated elevated production of fibroblast growth factor (FGF), interleukin 1 (IL1), platelet-derived growth factor (PDGF), and colony-stimulating factor (CSF) [39]. All of these findings support the assumption that these macrophages are activated to secrete a variety of cytokines associated with regenerative processes, as illustrated in Step 4 in Figure 2B.

## 7. Accelerated Scar-Free Regeneration of α-Gal Nanoparticle-Treated Wounds

**Figure 5:**
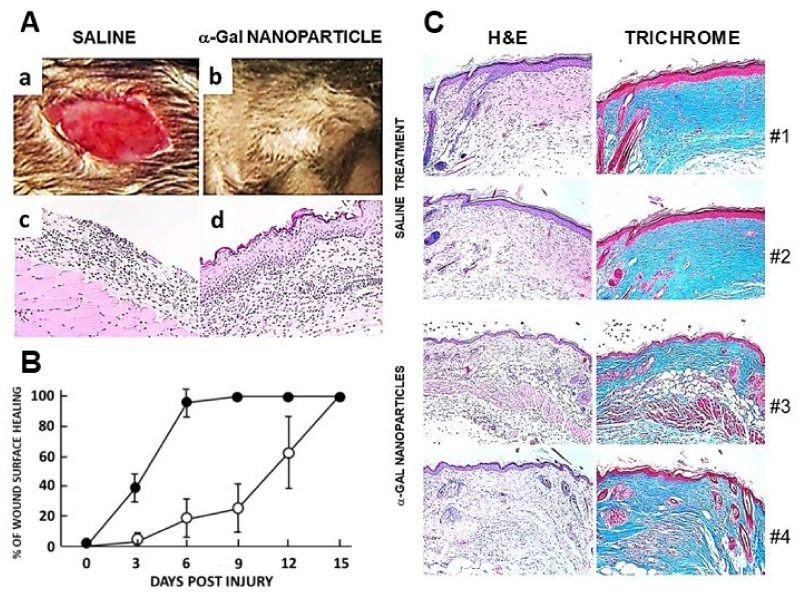
Accelerated scar-free regeneration of α-gal nanoparticle-treated skin wounds. (**A**) Representative wounds 6 days following treatment with saline or with 10 mg α-gal nanoparticles. (**a**,**b**) Gross morphology of excisional, full-thickness oval skin wounds (9 × 6 mm). (**c**,**d**) Histology of the wounds indicating that saline-treated wounds are not covered by regenerating epidermis, whereas α-gal nanoparticle-treated wounds are covered by such epidermis (H&E × 100). (**B**) Extent of wound healing at various days post-treatment with α-gal nanoparticles (●) or with saline (○), as measured by % of regenerating epidermis covering the wound. Note that most wounds treated with α-gal nanoparticles are completely healed by day 6, whereas healing of the saline-treated wounds takes 12–15 days (n = 5–8 mice per group except for day 6, in which n = 20, mean ± SD). (**C**) Day 28 histology of representative wounds treated with saline (#1 and #2) and wounds treated with α-gal nanoparticles (#3 and #4). The saline-treated wounds display fibrosis and scar formation characterized by thick epidermis and dense connective tissue (blue color in Mason Trichrome staining) in the dermis, with no hair shafts, adipocytes or smooth muscle cells. In contrast, wounds treated with α-gal nanoparticles are scar-free with thin epidermis, loose connective tissue in the dermis, hair shafts, adipocytes and smooth muscle cells as in healthy skin (×100). Adapted from Ref. [88] with permission.

The in vivo effects of the α-gal nanoparticles, postulated in Figure 2B, were first studied in wound and skin burn injuries in anti-Gal-producing GT-KO mice. Full-thickness dorsal excisional oval wounds (~9 × 6 mm) were performed in the mice. α-Gal nanoparticles (10 mg in saline) were applied to the wounds on wound dressings. Control wound dressings contained only saline. Extensive recruitment of macrophages into wounds treated with α-gal nanoparticles was observed by day 3, whereas control wounds lacked macrophages at that time point [39,88]. By day 6, most of the nanoparticle-treated wounds were covered with regenerating epidermis, whereas control wounds displayed macrophage recruitment but no significant epidermal regeneration (Figure 5A). Whereas most of the wounds treated with α-gal nanoparticles healed within 6 days, saline-treated wounds healed only after 12–15 days (Figure 5B). A similar accelerated healing was observed in skin burns [38,88]. Histology of the saline-treated wounds after 28 days revealed repair by fibrosis and scar formation, characterized by the thickening of both the epidermis and dermis. This dermis contained dense connective tissue (deep blue color of the collagen by Mason Trichrome staining), multiple fibroblasts, and no skin appendages such as hair shafts, sebaceous glands, smooth muscle cells, or adipocytes (Figure 5C, #1 and #2) [39]. In contrast, histology of α-gal nanoparticle-treated wounds, inspected after 28 days, demonstrated regeneration of the normal skin structure, including a normal thin epidermis and dermis. The dermis contained loose connective tissue, hair shafts, smooth muscle cells, and adipocytes (Figure 5C, #3 and #4). These findings imply that the treatment of wounds with α-gal nanoparticles modulated the healing mechanism from the default fibrosis and scar formation to accelerated healing that resulted in scar-free regeneration of the injured skin.

## 8. Post-MI Regeneration of Adult Mouse Heart by α-Gal Nanoparticles

MI in adult anti-Gal-producing mice was created by occlusion of the LAD with ligation at its mid-level for 30 min followed by reperfusion. Extensive infiltration of macrophages into the injured tissue was observed after 4 days. These macrophages disappeared within 7 days. After 14 days, the ventricular wall displayed thinning and fibrotic tissue appeared (Figure 1). In view of the accelerated healing and scar-free regeneration of skin wounds, it was of interest to determine whether α-gal nanoparticle treatment of post-MI injured myocardium can induce similar scar-free regeneration.

The heart of these mice was surgically exposed, and the mid-left LAD coronary artery was occluded by ligation for 30 min to simulate MI. Subsequently, the ligature was removed, allowing for reperfusion of the ischemic myocardium as in Figure 1. However, after ~3 min following reperfusion, the injured myocardium received two 10 µL injections each of 0.1mg α-gal nanoparticles in saline, ~2 mm distance of both sites from the ligation site. The chest was subsequently closed. Control mice underwent similar treatment but received two injections of 10 µL saline instead of the nanoparticles. The hearts were retrieved 4, 7, 14 and 28 days post-MI.

**Figure 6:**
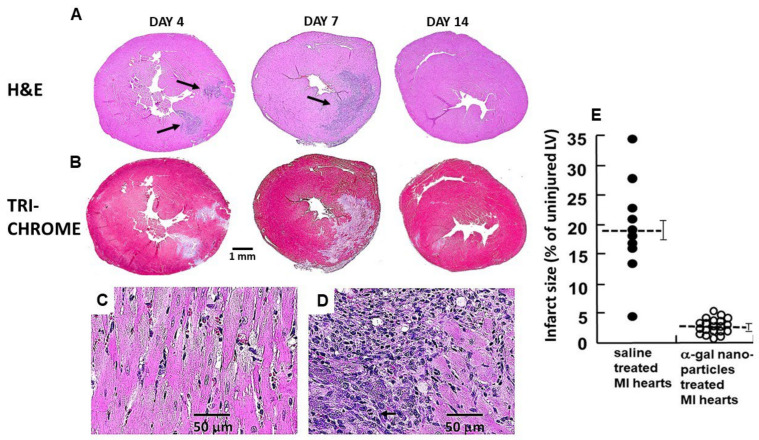
Representative post-MI regeneration of the left ventricular wall in adult mouse hearts treated with α-gal nanoparticles. The sections are stained with H&E (macrophages as purple dots in (**A**) or with Mason Trichrome staining (cardiomyocytes—red, fibrosis—grey and blue, and debrided areas—light pink or white in (**B**). Day 4: Macrophages infiltrate the areas that were injected with α-gal nanoparticles (marked by arrows). Day 7: Infiltration of macrophages peaks and the infiltrated areas are debrided. Day 14: The macrophages disappear and nearly all of the debrided area is replaced by regenerating cardiomyocytes. (**C**) Normal myocardial tissue (×400). (**D**) Three types of cells observed in day 7 sections: Normal cardiomyocytes in the right half of the figure, as those in (**C**), recruited macrophages in the upper left quarter, and cells larger than the macrophages but smaller than cardiomyocytes, in the lower left quarter. These cells display basophilic cytoplasm, suggesting proliferation of the cells. A clear mitotic figure among these cells is marked by an arrow (×400). (**E**). Comparison of left ventricular wall areas undergoing fibrosis in post-MI saline and α-gal nanoparticle-treated hearts, as measured by planimetry and presented as a proportion (%) of the uninjured left ventricle myocardium in 10 saline controls, and 20 α-gal nanoparticle-treated hearts (mean—dashed lines ± SE). Adapted from Ref. [34] with permission.

Histological evaluation of the hearts in α-gal nanoparticle-treated mice (Figure 6) revealed significant differences from that of saline-treated mice (Figure 1). The extent of macrophage infiltration in α-gal nanoparticle-treated hearts on day 4 was much lower than that in saline-treated hearts and the macrophages were found mainly around the two nanoparticles injection sites, as indicated in H&E staining (Figure 6A). In contrast, on day 7, the nanoparticle-treated hearts contained many more macrophages than on day 4, whereas in saline-treated hearts, almost all macrophages disappeared by day 7. It is of note that in both treated and control hearts, the lack of Masone Trichrome staining in areas infiltrated by macrophages on days 4 and 7 strongly suggests that the macrophages debrided in areas where they resided (Figure 1 and Figure 6B, respectively), like the debriding by macrophages observed in regenerating urodele hearts [16]. It is possible that the observed debriding by macrophages in both regenerating myocardium in Figure 6B and in the reparative processes leading to fibrosis and scar formation in Figure 1 are mediated by metalloproteinases such as MMP9. This protease was found in single-cell transcriptomic studies to be secreted by macrophages, and it destroys collagen in cardiac allograft vasculopathy [89].

Histology sections on day 7 of injured myocardial wall treated with α-gal nanoparticles reveal the presence of two types of cells which are not cardiomyocytes (Figure 6D). The small mononuclear cells display the morphology of infiltrating macrophages, and the larger cells (smaller than cardiomyocytes) seem to be proliferating cells (see mitotic figure), larger than macrophages. The identity of these cells requires further investigation.

**Figure 7:**
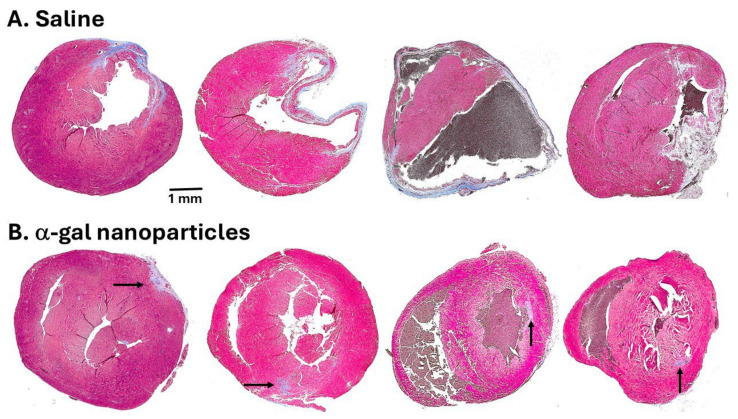
Comparative histology of myocardial repair and regeneration of saline (**A**) and α-gal nanoparticle-treated hearts (**B**) as viewed on day 28 post-MI. The grey-blue color in the Mason Trichrome staining identifies fibrotic tissue in the scars of saline-treated left ventricular walls and the residual fibrosis in α-gal nanoparticle-treated left ventricular walls (marked by arrows). Adapted from Ref. [34] with permission.

By day 14, the saline-treated hearts displayed thinning of the left ventricular wall and initial indications of fibrosis and scar formation (observed in Masone Trichrome staining in Figure 1). In contrast, α-gal nanoparticle-treated hearts displayed regeneration of the injured myocardium on that day (Figure 6B). The differences between saline and α-gal nanoparticle-treated hearts increased in specimens analyzed on day 28 post-MI (Figure 7). Hearts injected with saline displayed distinct trans-myocardial fibrosis and scar formation, which in most hearts ranged between 15% and 35% of the left ventricular wall, with an average scar size of 19% (Figure 6E and Figure 7A). In contrast, α-gal nanoparticle-injected hearts displayed near-complete regeneration of the ventricular wall and only minimal indication of fibrosis, with an average scar size of 2% (Figure 6E and Figure 7B). The safety of this treatment is supported by the finding that more than 75% of the mice survived the initial surgical occlusion/reperfusion procedure. These mice remained alive through day 28, when final echocardiography and euthanasia were performed. No granulomas were observed on either day 14 or day 28 post-treatment.

**Figure 8:**
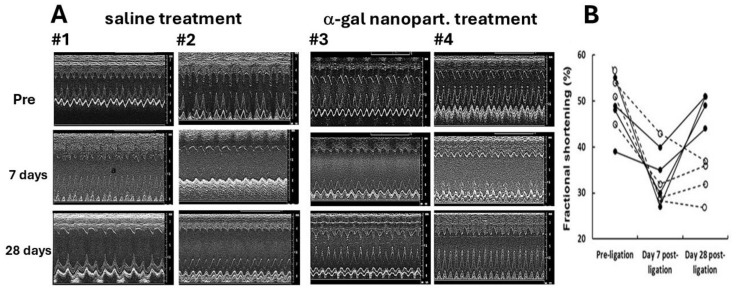
Restoration of contractility in post-MI heart treated with α-gal nanoparticles, as determined by the following: (**A**) Echocardiography and (**B**) fractional shortening calculations. (**A**) Echocardiography of saline-treated (#1 and #2) and of α-gal nanoparticle-treated hearts (#3 and #4) at three time points: pre-LAD ligation, 7 days post-ligation and 28 days post-ligation. (**B**) Fractional shortening at the three time points in four mice evaluated in saline-treated (○—and dashed lines) and α-gal nanoparticle-treated hearts (●—and solid lines). Data presented as percentage changes in fractional shortening at pre-ligation, 7 days post-ligation and 28 days post-ligation. Adapted from Ref. [34] with permission.

Functional regeneration of the α-gal nanoparticle-treated injured myocardium could be demonstrated by echocardiography (Figure 8). In both saline-treated and α-gal nanoparticle-treated mice, the left ventricular function prior to ligation of the LAD demonstrated normal fractional shortening of ~50%. Due to the ischemia injury, the fraction shortening in both groups on day 7 post-MI was only ~35%, associated with chamber dilation and correlated with the histopathology of the left ventricles at that day (Figure 1 and Figure 6). A similar decreased level of fractional shortening was also measured on day 28 in the saline-treated mice (Figure 8A,B) due to the irreversible damage of the left ventricular wall (Figure 7A). However, in the α-gal nanoparticle-treated mice, the fractional shortening increased on day 28, recovering to ~50% (Figure 8A,B), which correlated with the regeneration of the injured myocardium (Figure 7B).

The mechanism of regeneration in α-gal nanoparticle-treated injured hearts in adult mice is not fully understood. The different outcomes in injured hearts injected with saline and those injected with α-gal nanoparticles (Figure 7) imply that the pro-reparative macrophages infiltrating into saline-treated myocardium mediate the default repair mechanism of fibrosis and scar formation, like in wound healing. In contrast, the pro-regenerative macrophages recruited by α-gal nanoparticles mediate regeneration processes (Figure 6A) like those observed in scar-free wound healing (Figure 5A-d).

The characteristics of pro-regenerative macrophages are not clear at present. In accord with a previous suggestion [90] regarding M1/M2 polarization, these markers are not applicable to the pro-regenerative vs. pro-reparative macrophages since both groups are characterized as M2 macrophages (see Figure 4B and Refs. [29,83,91]). It would be of interest to determine whether they display characteristics of two macrophage markers: (1) Embryonic-derived cardiac macrophages which lack the CC-motif chemokine receptor 2 (CCR2). The CCR2^-^ are tissue-resident macrophage populations involved in tissue development, repair and maintenance, whereas CCR2^+^ macrophages are derived from circulating monocytes and participate in inflammation processes and tissue damage [91,92]. (2) T-cell immunoglobulin and mucin domain-containing 4 (TIMD-4). TIMD-4^+^ are resident macrophages in the heart, whereas TIMD-4^−^ macrophages are derived from recruited monocytes [92].

The rapid transition of the macrophage-debrided areas in the α-gal nanoparticle-treated hearts of day 7, to full replacement with normal myocardium tissue on day 14 (Figure 6A,B), suggests extensive proliferation of cells that differentiate into cardiomyocytes. It remains to be determined whether the proliferating cells are of recruited mesenchymal stem cells, precursor cardiac cells, or dedifferentiated cardiomyocytes. Nevertheless, the results of post-MI treatment with α-gal nanoparticles clearly suggests that these nanoparticles induce a sequence of processes that lead to near complete regeneration of the injured myocardium.

It is difficult to compare the near-complete to complete regeneration of post-MI mouse hearts treated with α-gal nanoparticles which induce polarization of macrophages into a pro-regenerative state (Figure 6, Figure 7 and Figure 8) with other methods studied for inducing regeneration of injured myocardium by activating cardiomyocytes to proliferate. Some of the latter methods include modulation of oxygen level [93], changing energy levels by upregulating glycolysis [94], activation of cell cycle-associated genes for induction of cardiomyocyte cell division [94,95], activation of proliferative signaling pathways [92,96,97,98,99], glucocorticoids [100], progesterone for enhancement of cardiomyocyte proliferation through the upregulation of yes-associated protein (YAP) expression [101], activation of various transcription factors, and use of miRNA [102,103]. The difficulty in such comparison stems from the fact that most of these method report “improvement of function” whereas the α-gal therapy measures 95–100% regeneration. Intra-myocardial administration of mesenchymal stem cells was also studied for inducing regeneration of injured myocardium but was found to display suboptimal efficacy. The main obstacle was found to be associated with reduced cell retention and low engraftment [104,105].

In addition to the need for better understanding of the mechanisms involved in the regeneration process, studies are required for establishing the efficacy of the α-gal nanoparticle treatment in a large animal model which may better simulate the possibility of performing this treatment in post-MI patients. A practical animal model is the knockout pig for the α1,3GT gene (GT-KO pig). This engineered pig lacks α-gal epitopes [106,107] and produces the natural anti-Gal antibody as well as humans [108,109]. Simulation of MI in these pigs can be performed by temporarily blocking the LAD, followed by reperfusion, and trans-endocardial administration of α-gal nanoparticles with a catheter. Success in anti-Gal mediated regeneration by α-gal nanoparticle treatment in occlusion/reperfusion studies in GT-KO pigs will further enable the determination of how many hours after the infarction is the window of time available for enabling an effective induction of regeneration, instead of irreversible fibrosis and scar formation. Success in such studies also may enable the initiation of planning for this treatment in post-MI patients. The fact that anti-Gal is produced in all humans who are not severely immunocompromised, including in individuals above the age of 75 [44], suggests that harnessing of this antibody may be feasible at any age.

Production of large amounts of α-gal nanoparticles is feasible by synthesizing synthetic α-gal glycolipids either by chemical reactions [110] or by enzymatic reactions with recombinant α1,3GT [111,112,113]. A few clinical trials in cancer patients were performed with tumor cells engineered to present multiple α-gal epitopes, aiming to convert these cells into vaccines against autologous tumor antigens. These trials reported that exposure of the immune system to α-gal epitopes is safe and has no significant adverse side effects in treated patients [111,112,113,114].

Overall, the findings in this review suggest that treatment of post-MI-injured myocardium in anti-Gal producing adult mice with α-gal nanoparticles reactivates, by an as-yet-unknown mechanism, the innate regenerative mechanism that is suppressed within a few days after the birth of mice, thus restoring most of the structure and function of the heart.

## 9. Concluding Remarks 

The ability to regenerate injured myocardium seems to be an innate mechanism that appeared in early stages of vertebrate evolution. This innate mechanism is evident in zebrafish and in salamander, newt and axolotl. The demonstration of such innate regeneration during the first days after the birth of mice and pigs raised the possibility that this ancient regenerative mechanism was conserved in mammals as well. However, the repair mechanism in post-MI injured myocardium in adult mammals results in fibrosis and scar formation in the ischemic tissue. This age-associated change implies that the active regenerative mechanism in neonates is suppressed within several days after birth and replaced by the fibrosis and scar formation repair mechanism. The common phenomena of early macrophage infiltration into injury sites and activation of parts of the complement system in the innate regeneration processes further raised the possibility that localized activation of the complement system for induction of macrophages recruitment in post-MI adult mouse heart may reactivate the regenerative mechanism which is suppressed in early mammalian life. This activation could be achieved by administration of α-gal nanoparticles into the post-MI ischemic myocardium of anti-Gal-producing adult mice. This natural antibody, which constitutes ~1% of human immunoglobulins, binds to the multiple α-gal epitopes on α-gal nanoparticles and activates the complement system within the treated injured tissue. The complement cleavage chemotactic peptides C5a and C3a induce extensive recruitment of macrophages, which bind and internalize the anti-Gal-coated α-gal nanoparticles via Fc/Fcγ receptor and C3b/CR1 interactions. These processes further induce polarization of the recruited macrophages into pro-regenerative macrophages which orchestrate regeneration of the ischemic myocardium into normal contractile tissue, instead of the fibrosis and scar formation observed in control post-MI heart healing. A similar scar-free regeneration occurs in skin wounds of anti-Gal-producing adult mice treated with α-gal nanoparticles. The molecular mechanisms mediating the observed cardiac regeneration in the adult mice require further investigation. Nevertheless, the observed regenerative processes in adult mouse heart and skin suggest that the α-gal nanoparticles/anti-Gal interaction may provide a novel regenerative therapy in adult mammals that recapitulates the innate regenerative processes in fish, urodeles and mammalian neonates. Future evaluation of this approach in larger animal models will determine the translational potential for clinical application in acute myocardial infarction.

## Data Availability

No new data were created or analyzed in this study.

## Abbreviations

The following abbreviations are used in this manuscript:

α-gal	carbohydrate antigen with the structure Galα1-3Galβ1-4GlcNAc-R
α1,3GT	α1,3galactosyltransferase
CR1	complement receptor 1
GT-KO	mouse or pig knockout for the α1,3galactosyltransferase gene (*GTTA1*)
LAD	left anterior descending
MI	myocardial infarction
PVA	polyvinyl alcohol
